# Self-rated health status and associated factors in Ilam, west of Iran: results of a population-based cross-sectional study

**DOI:** 10.3389/fpubh.2024.1435687

**Published:** 2025-01-07

**Authors:** Mohammad Bazyar, Hojatollah Kakaei, Hamed Azadi, Mohsen Jalilian, Mohammad Ali Mansournia, Kamran Malekan, Reza Pakzad

**Affiliations:** ^1^Department of Health Management and Economics, Faculty of Health, Ilam University of Medical Sciences, Ilam, Iran; ^2^Department of Occupational Health, Faculty of Health, Ilam University of Medical Sciences, Ilam, Iran; ^3^Department of Anesthesiology, School of Allied Medical Sciences, Ilam University of Medical Sciences, Ilam, Iran; ^4^Department of Public Health, Faculty of Health, Ilam University of Medical Sciences, Ilam, Iran; ^5^Department of Epidemiology, School of Public Health, Tehran University of Medical Sciences, Tehran, Iran; ^6^School of Medicine, Kurdistan University of Medical Sciences, Sanandaj, Iran; ^7^Health and Environment Research Center, Ilam University of Medical Sciences, Ilam, Iran; ^8^Psychosocial Injuries Research Center, Ilam University of Medical Sciences, Ilam, Iran; ^9^Department of Epidemiology, Faculty of Health, Ilam University of Medical Sciences, Ilam, Iran; ^10^Student Research Committee, Ilam University Medical Sciences, Ilam, Iran

**Keywords:** self-rated health, cross sectional study, prevalence, logistic models, risk factors

## Abstract

**Background:**

Self-rated health (SRH) is a single-item subjective indicator that asks individuals to assess their overall health and acts as a good indicator to reveal general health status. This study aimed to determine the SRH status and determining factors.

**Methods:**

This was a population-based cross sectional study conducted in Ilam city (West of Iran) in 2023. A total of 1,370 people were invited to participate in the study using multi-stage stratified cluster random sampling method. Demographic and SRH status data were collected by face-to-face interview. SRH was indicated by a single question in five scales of very good, good, fair, poor and very poor. Multiple ordinal logistic regression was used for data analysis.

**Results:**

The 59.38% (95% CI: 56.76 to 62) participants reported a good SRH status. By ordinal multiple logistic regression, odds ratio (OR) and 95% confidence interval (CI) was calculated and based on that, female gender [OR: 1.68 (1.29 to 2.20)], not having insurance coverage [OR: 1.35; (1.01 to 1.80)], history of job loss [OR: 1.72; (1.28 to 2.31)], hopelessness for the future [OR: 5.07; (3.96 to 6.49)], and having underlying diseases [OR: 2.95; (2.25 to 3.88)], were positively associated with poor SRH status. The Kurd race [OR: 0.45; (0.25 to 0.78)], higher economic status [OR: 0.72; (0.54 to 0.96)] and use of health care service [OR: 0.68; (0.53 to 0.88)] were negatively associated with poor SRH status. The most effective variables for poor SRH status were hopelessness about the future and suffering from underlying diseases.

**Conclusion:**

It is important to devise corrective measures and effective public health policies to address causes and factors associated with poor SRH. It is also necessary for local health officials to allocate financial resources and introduce other kinds of supportive initiatives to provide targeted support for those who are struggling with poverty and suffering chronic diseases.

## Introduction

Self-rated health (SRH) (also known as health perception or perceiving health), is a single-item subjective indicator which aims to assess general health status of individuals (1, 2). SRH is a simple question requesting individuals to assess their overall health relatively. They may value their health status in comparison with the health of other people with the same age in the society (3).

As SRH is a subjective indicator, it could be influenced by many various physical and intellectual factors. For example, according to previous studies, some of determining factors include socioeconomic status, level of education (4), perceived family respect, (5), attitudes and satisfaction of individuals about their appearance and weight and also gender inequalities, (6, 7); geographical aspects such as residential differences (8); demographic features and current health problems including age, number of diseases; and lifestyle behaviors such as physical exercise (9).

Recent evidence suggests that the strength of association between SRH status and a higher risk of developing chronic diseases later in life, such as diabetes, lung disease, coronary heart disease, arthritis, and stroke (10, 11), may vary across countries (12). This variability could also apply to other health aspects that warrant further investigation. Additionally, cultural backgrounds have been shown to significantly shape individuals’ perspectives on health (1, 13). Therefore, more studies from diverse contexts are needed to gain a comprehensive understanding of determining factors affecting SRH. The decision to conduct this study in Ilam province, Iran, was primarily based on two reasons. Firstly, Iranian people have been facing a severe economic condition and the COVID-19 pandemic in recent years which may have affected all aspects of their lives. Secondly, to best of the authors’ knowledge, no previous studies have been conducted among people living in Ilam.

### Study context

Ilam province possesses unique characteristics which make it a compelling location for health related research. Ilam province, has suffered chronically from poor economic and developmental indicators (14, 15). With a mere 0.99% contribution to the national GDP as of 2016, Ilam ranks as one of Iran’s least developed provinces, coming in at 26th out of 31. Ilam province is among the regions with lowest population, with 580,158 people according to the 2016 census. Located in the west of Iran, it is the neighbor of Iraq with 425 km where about 70 percent of people speak Kurdish (16, 17). Our objective was to investigate the prevalence of poor self-rated health (SRH) and to gain new insights into the factors influencing SRH in the Kurdish region. This can lead to a deeper understanding of the SRH state. Comparing and combining of various findings from wide range of studies can help policy makers to make reliable decisions based on scientific information. This in turn can help decision makers to adapt both general and region-specific policies to combat the main contributors of poor SRH.

## Methods

### Setting and study design

**Figure 1:**
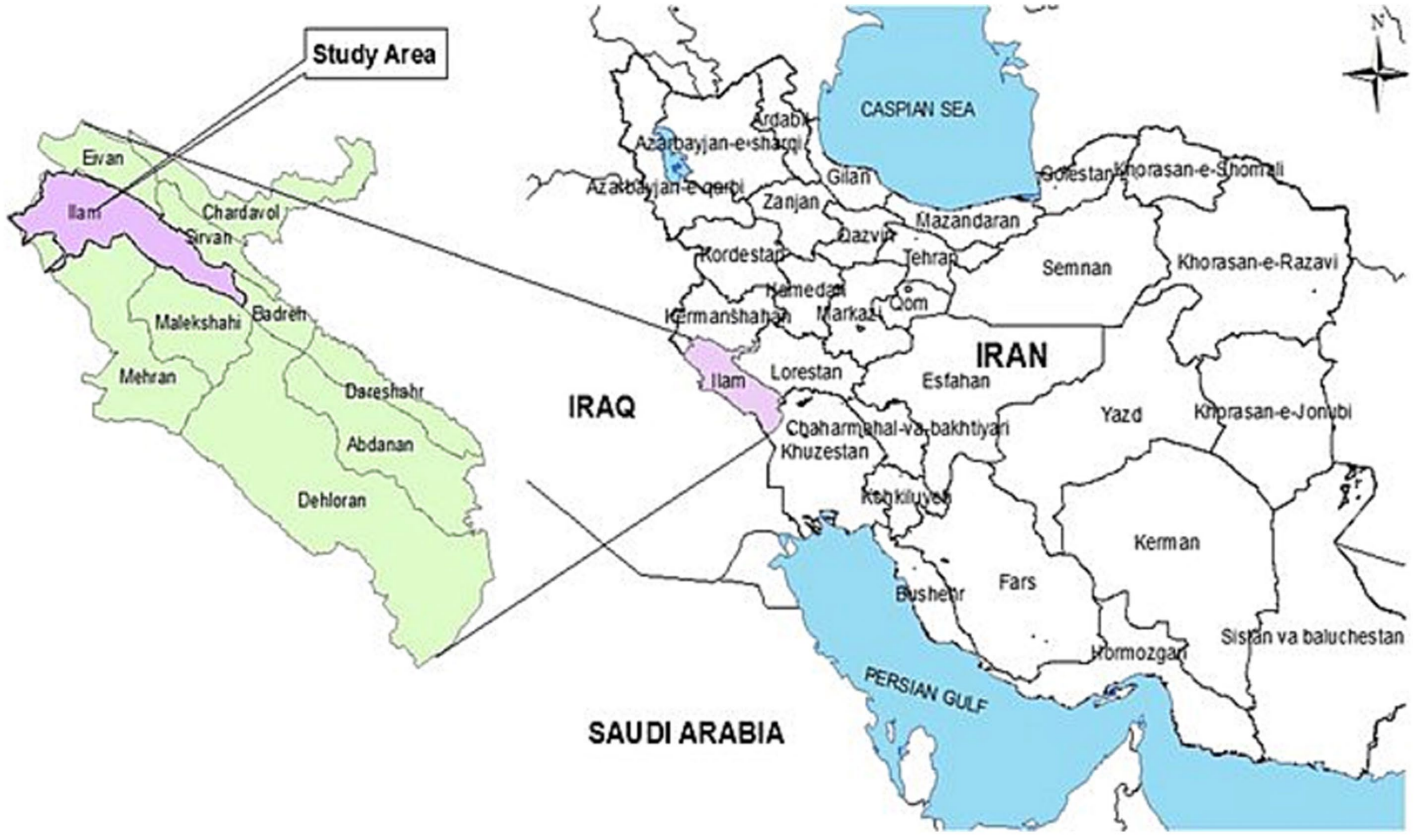
Geographical location of Ilam city (the figure has been generated by the authors).

This was a secondary data analysis that was done on previous gathered data. The methodology of the present study was published elsewhere and is briefly explained here (18). This population-based cross-sectional study was conducted in Ilam, Iran, which has a population of 201,000 people. All people over 15 years old was considered as target population. Ilam is the least populated province in Iran. The geographical location of Ilam city can be seen in Figure 1.

### Sample size and sampling

According to Maharlouei et al.’s study in Shiraz, the rate of good-SRH was 47.3% (10). With a confidence level 95% and the precision = 0.05, the sample size was estimated to be 381 using the following formula.


$$n=\frac{z^{2}pq}{d^{2}}=\frac{1.96^{2}*0.54*0.46}{0.05^{2}}=381$$

However, considering the type of sampling and a design effect of 2.5, the estimated sample size was 952. Taking into account an attrition rate of 10% and missing data rate of 20%, the minimum sample size was determined to be 1,230. The participants were selected using a multi-stage stratified cluster random sampling approach with a proportion-to-size method. Ilam city was divided into 10 areas based on the locations of 10 health centers, and the population served by each area was identified. Then, cluster sampling was used to select groups within each area, with each group consisting of 20 people. The sampling team began sampling households in each group by moving counterclockwise from the southwest corner of the group until the desired sample size for that group was reached.

Inclusion and exclusion criteria: All family members over the age of 15 were invited to participate after being informed about the study objectives and ensuring the confidentiality and anonymity of their data. However, people not willing to participate in the study, people with cognitive diseases and those who could not understand the questions correctly were excluded from the study.

### Data collection

Interview guideline is published elsewhere (18) and provided as Supplementary material S1. A 22 item research-made questionnaire was developed by authors and each questions was scaled by related answer. Description of study variables was provided in Supplementary Table S1 and definitions with coding was highlighted. Data was gathered through face-to-face structured interviews with knowledgeable interviewers. The collected data included demographic information such as age (in years), gender (female and male), race (Kurd and other), education level (below diploma, diploma-associate degree and bachelor’s degree or higher), household size (number of members), occupation (student, employed and retired), health insurance coverage (covered, un-covered), economic status (assessed through a principal component analysis of 15 questions related to assets including ownership of cars, motorcycles, refrigerators, washing machines, macro-waves, laptops, vacuums, dishwashers, internet access, LCD TVs, DVD players, going to restaurants, traveling, house, and steam irons), and other variables such as co-morbidities (diabetes, hypertension, asthma, cancer, central nervous disorder, musculoskeletal disorder, cerebrovascular diseases and kidney disease), mental disorders history, family member death history (Yes or No), job loss history (Yes or No), and participants’ future hopes (assessed using a five-point Likert scale question: “In general, considering the current situation, how hopeful are you for the future?“).The smoking (have you ever smoked cigarettes in your lifetime? Yes, or No), Alcohol (have you ever consumed alcohol in your lifetime? Yes, or No), and Hookah (have you ever smoked hookah in your lifetime? Yes, or No) were evaluated by youth risk behavior surveillance questionnaire (YRBSQ) whom validated (Cronbach’s alpha for different dimensions ranging from 78.4 to 94.1%) by Baheiraei et al. (19).

The dependent variable in this study was SRH, which was assessed using the question “In general, how would you rate your health?” with response options on a Likert-scale ranging from very good to very poor. The responses of very good and good were combined into the category of “good SRH status,” fair remained as “fair SRH status,” and the responses of poor and very poor were combined into the category of “poor SRH status.” The validity and reliability of this tool were previously confirmed by Maharlouei et al. (10).

### Statistical analysis

To estimate the age and sex-standardized rate of SRH, the sample taken was weighted based on the Ilam city population. Next, the age-sex standardized rate of SRH was estimated with 95% confidence interval. Initially, a simple ordinal logistic regression was conducted to examine the relationship between the studied outcomes and demographic variables. Subsequently, a multiple ordinal logistic regression was employed to construct a full model and assess the simultaneous impact of study variables on the outcomes. It is important to note that variables were included in the multiple ordinal logistic regression model based on a significance level of less than 0.05. For checking proportionality of odds, a likelihood-ratio test was used and multicollinearity was checked by Variance Inflation Factor (VIF). Those variables with VIF more than 10 was considered as collinear variables and was excluded from model. Also the cluster effect was taken into account to correct for sampling error. The standardized coefficients were used to identify the most significant variables in the full model. All analyzes were performed using Stata version 12, with a significance level of 0.05.

## Results

A total from a total of 1,370 participants were included in the analysis. The demographic and other characteristics of the participants can be seen in pervious study (18). The mean age of the participants was 40.45 years, and 50.30% of the participants were female. In terms of SRH status 814 (59.38%) of the participants reported good SRH, 435 (31.76%) reported fair SRH and only 121 (8.86%) had poor SRH.

Table 1 presents the rate of SRH status based on study variables and the results of simple ordinal logistic regression. Female gender, lower educational level, being a housekeeper or unemployed, lack of insurance coverage, job loss history, history of mental disorder, hopelessness for the future, and losing family members were all associated with a higher likelihood of poor SRH status. On the other hand, Kurd race, high economic status and use of healthcare services were associated with a lower likelihood of poor SRH status.

**Table 1:** SRH status and influencing variables using simple ordinal logistic regression.

Variables		SRH				
		N (%)			Ordinal logistic regression	
		Good	Fair	Poor	OR (95% CI)	*p*-value
Gender	Male	426 (62.65)	202 (29.71)	52 (7.65)	Reference	---
	Female	388 (56.23)	233 (33.77)	69 (10)	1.31 (1.06 to 1.62)	0.012*
Race	Other	56 (71.79)	15 (19.23)	7 (8.97)	Reference	---
	Kurd	758 (58.67)	420 (32.51)	114 (8.82)	0.59 (0.36 to 0.98)	0.040*
Education level	< Diploma	210 (49.76)	153 (36.26)	59 (13.98)	Reference	---
	Diploma & Associate Degree	267 (58.42)	154 (33.7)	36 (7.88)	0.67 (0.52 to 0.87)	0.002*
	≥ Bachelor	337 (68.64)	128 (26.07)	26 (5.3)	0.44 (0.33 to 0.56)	<0.001*
Job	Student	138 (71.5)	44 (22.8)	11 (5.7)	Reference	---
	Employed	156 (72.22)	51 (23.61)	9 (4.17)	0.94 (0.61 to 1.45)	0.804
	Retrieved	79 (51.63)	59 (38.56)	15 (9.8)	2.27 (1.47 to 3.50)	<0.001*
	Housekeeper or unemployed	441 (54.58)	281 (34.78)	86 (10.64)	2.09 (1.49 to 2.93)	<0.001*
Insurance coverage	Yes	636 (61.21)	326 (31.38)	77 (7.41)	Reference	---
	No	178 (53.78)	109 (32.93)	44 (13.29)	1.43 (1.12 to 1.83)	0.004*
Economic status	Low	247 (48.34)	190 (37.18)	74 (14.48)	Reference	---
	Middle	270 (62.79)	136 (31.63)	24 (5.58)	0.52 (0.40 to 0.67)	<0.001*
	High	297 (69.23)	109 (25.41)	23 (5.36)	0.40 (0.31 to 0.52)	<0.001*
Job loss history	No	671 (61.9)	335 (30.9)	78 (7.2)	Reference	---
	Yes	143 (50)	100 (34.97)	43 (15.03)	1.73 (1.34 to 2.23)	<0.001*
Mental disorder History	No	772 (61.42)	383 (30.47)	102 (8.11)	Reference	---
	Yes	42 (37.17)	52 (46.02)	19 (16.81)	2.57 (1.79 to 3.69)	<0.001*
Hope for the future	Yes	606 (75.47)	178 (22.17)	19 (2.37)	Reference	---
	No	208 (36.68)	257 (45.33)	102 (17.99)	5.63 (4.47 to 7.08)	<0.001*
Underlying diseases	No	629 (70.59)	227 (25.48)	35 (3.93)	Reference	---
	Yes	185 (38.62)	208 (43.42)	86 (17.95)	4.02 (3.21 to 5.04)	<0.001*
Loss of family members	No	464 (65.72)	199 (28.19)	43 (6.09)	Reference	---
	Yes	350 (52.71)	236 (35.54)	78 (11.75)	1.76 (1.42 to 2.18)	<0.001*
Use of health care service	No	334 (55.67)	207 (34.5)	59 (9.83)	Reference	---
	Yes	480 (62.34)	228 (29.61)	62 (8.05)	0.76 (0.62 to 0.95)	0.013*
Have a doctor visit	No	202 (61.59)	100 (30.49)	26 (7.93)	Reference	---
	Yes	611 (58.69)	335 (32.18)	95 (9.13)	1.13 (0.88 to 1.45)	0.327
Smoking	No	753 (60.92)	380 (30.74)	103 (8.33)	Reference	---
	Yes	61 (45.52)	55 (41.04)	18 (13.43)	1.83 (1.30 to 2.57)	<0.001*
Alcohol consumption	No	792 (59.41)	422 (31.66)	119 (8.93)	Reference	---
	Yes	22 (59.46)	13 (35.14)	2 (5.41)	0.94 (0.49 to 1.80)	0.863
Hookah use	No	747 (59.76)	387 (30.96)	116 (9.28)	Reference	---
	Yes	67 (55.83)	48 (40)	5 (4.17)	1.05 (0.73 to 1.51)	0.760
Age (yrs. old)		38.44 (15.09)	42.65 (14.89)	46.07 (17.04)	1.03 (1.02 to 1.04)	<0.001*
Family size (number of members)		4.21 (1.57)	4.34 (1.57)	4.10 (1.67)	1.02 (0.96 to 1.09)	0.536
BMI (Kg/M^2^)		25.43 (3.91)	26.31 (4.44)	26.43 (4.81)	1.05 (1.03 to 1.08)	<0.001*

^*^Significant at 0.05 level. BMI: Bod Mass Index; SRH: Self-Rated Health.

**Figure 2:**
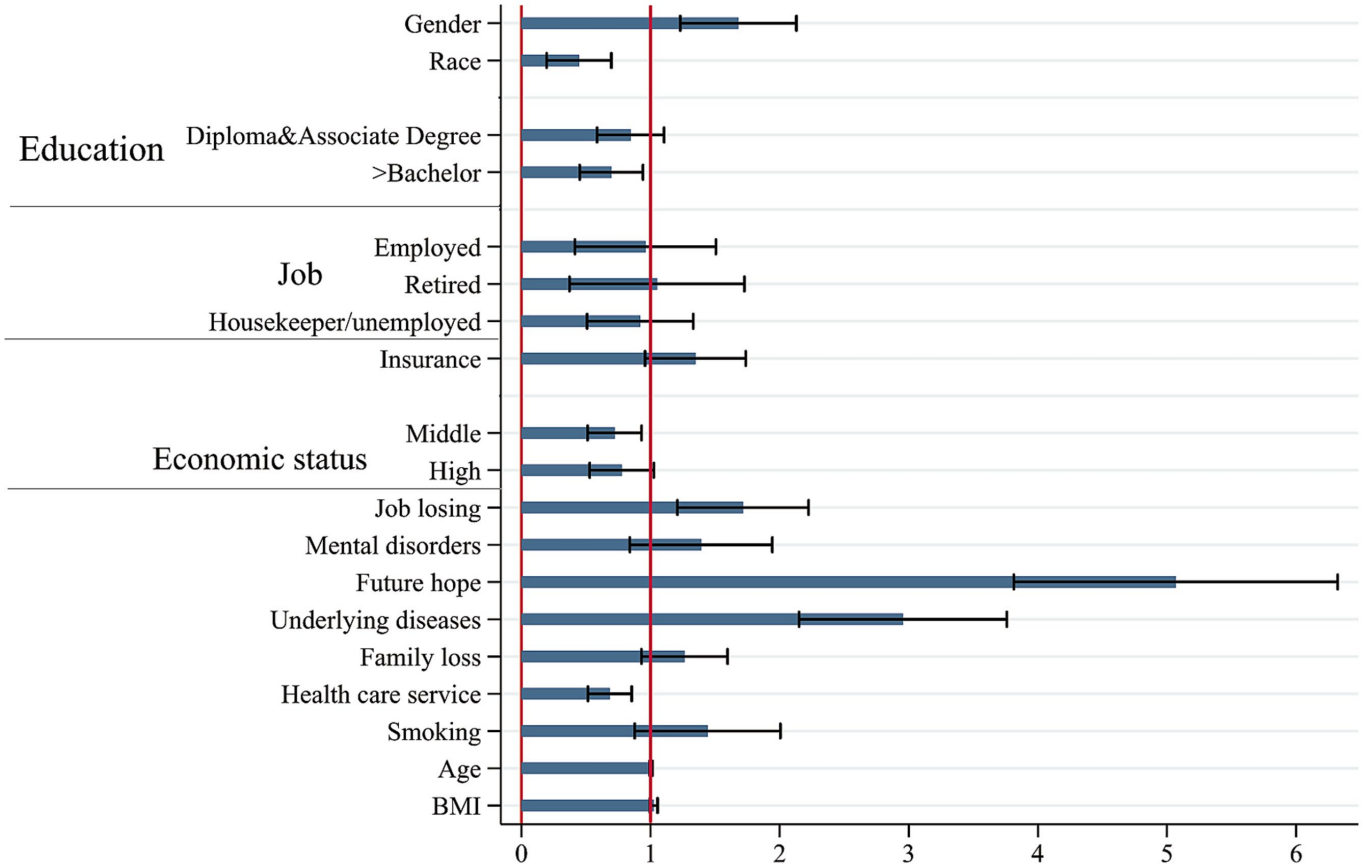
Visual results of multiple ordinal logistic regression model for association between SRH with determinants.

As stated in the methodology section, a multiple ordinal logistic regression was conducted to assess the simultaneous impact of the study variables. To do so, variables with a significance level of less than 0.05 were included in the full model. Table 2 presents the results of the multiple ordinal logistic regression model. Adjusted for the included variables, the female gender (OR: 1.68; 95% CI: 1.29 to 2.20; *p* < 0.001), lack of insurance coverage (OR: 1.35; 95% CI: 1.01 to 1.80; p:0.044), job loss history (OR: 1.72; 95% CI: 1.28 to 2.31; *p* < 0.001), hopelessness about the future (OR: 5.07; 95% CI: 3.96 to 6.49; *p* < 0.001), and underlying diseases (OR: 2.95; 95% CI: 2.25 to 3.88; *p* < 0.001) were positively associated with a poor status of self-rated health (SRH). On the other hand, being of Kurdish race (OR: 0.45; 95% CI: 0.25 to 0.78; p: 0.005), having an educational level higher than bachelor’s degree (OR: 0.71; 95% CI: 0.49 to 0.99; p:0.044), having a middle economic status (OR: 0.72; 95% CI: 0.54 to 0.96; p:0.027), and utilization of healthcare services (OR: 0.68; 95% CI: 0.53 to 0.88; p: 0.003) were negatively associated with a poor status of SRH. Other significant variables in the simple model, such as job status, history of mental disorders, and loss of family members, did not have a significant effect in the multiple model. It should be noted that the proportionality of odds assumption was hold [chi2 (15) = 14.72; *p* = 0.472] and there was no collinearity between included variables. Figure 2 showed the visual results of multiple ordinal logistic regression model.

**Table 2:** The association between SRH status and study variables using multiple ordinal logistic regression.

Variables		SRH		VIF	Standardized coefficient
		OR (95% CI)	*p*-value		
Gender	Male	Reference	---	1.042	0.220
	Female	1.68 (1.29 to 2.20)	<0.001*		
Race	Other	Reference	---	1.570	−0.198
	Kurd	0.45 (0.25 to 0.78)	0.005*		
Education level	< Diploma	Reference	---	2.417	−0.171
	Diploma and associate degree	0.85 (0.62 to 1.15)	0.283		
	≥ Bachelor	0.70 (0.49 to 0.99)	0.044*		
Job	Student	Reference	---	1.714	Not included
	Employed	0.96 (0.54 to 1.70)	0.889		
	Retired	1.05 (0.55 to 2.01)	0.884		
	Housekeeper or unemployed	0.92 (0.59 to 1.44)	0.713		
Insurance coverage	Yes	Reference	---	3.478	0.106
	No	1.35 (1.01 to 1.80)	0.044*		
Economic status	Low	Reference	---	3.010	−0.137
	Middle	0.72 (0.54 to 0.96)	0.027*		
	High	0.78 (0.56 to 1.07)	0.122		
Job losing history	No	Reference	---	1.001	0.222
	Yes	1.72 (1.28 to 2.31)	<0.001*		
Mental disorder history	No	Reference	---	1.657	Not included
	Yes	1.39 (0.94 to 2.07)	0.103		
Hope for the future	Yes	Reference	---	2.978	0.807
	No	5.07 (3.96 to 6.49)	<0.001*		
Underlying diseases	No	Reference	---	3.009	0.590
	Yes	2.95 (2.25 to 3.88)	<0.001*		
Loss of family members	No	Reference	---	3.574	Not included
	Yes	1.26 (0.97 to 1.64)	0.083		
Use of health care service	No	Reference	---	4.587	−0.174
	Yes	0.68 (0.53 to 0.88)	0.003*		
Smoking	No	Reference	---	2.699	Not included
	Yes	1.44 (0.97 to 2.13)	0.067		
Age (Yrs. old)		1.01 (0.99 to 1.01)	0.526	3.230	Not applicable
BMI (Kg/M^2^)		1.02 (1.01 to 1.05)	0.099	1.032	Not applicable

^*^Significant at 0.05 level. BMI: Bod Mass Index; SRH: Self-Rated Health; VIF: Variance Inflation Factor.

One of the objectives of the study was to determine the most effective variables on SRH status. To achieve this, the standardized coefficients were estimated, and the results are presented in Table 2. The most influential variables for a poor status of SRH were found to be hopelessness for the future (0.807) and underlying diseases (0.590).

## Discussion

The findings showed that just less than 9 percent of participants assessed their health status as poor. This finding is consistent with a previous study conducted in Shiraz, southwest of Iran, in late 2014 (10) where a smaller proportion of individuals assessed their health as poor or bad with 5.9%. Although in another study conducted in Sanandaj in 2012 among female population, the proportion of individuals reporting poor SRH was as high as 37.6% (1). This rate of poor SRH in a similar study in US was 21% (20). A study conducted on homeless women in India showed that this figure can be high as 57% (21). This indicates that poor SRH in deprived and marginalized groups can be significantly higher studies done on general population. Furthermore the findings of this study and other studies conducted in from Iran (22) and India (23), revealed that women had a higher chance of reporting poor SRH status than men. It should be added that the people of Ilam, due to the weaker economic development in comparison with other regions of the country during the past decades, are likely to be more adaptable to the hardships of life, and this may reduce the chance of assessing their health as poor despite facing unprecedented excruciating economic condition over the last years which can affects their health in different ways. For example, hopelessness about the future was among the factors that negatively influenced self-rated health status. The high rate of hopelessness among people (37%) might have been affected by worse economic condition situation in Iran over the last years. Another factor contributing to the bad SRH was job loss. According to the findings, 20 percent of participants stated the history of job loss which is high. This in turn might contribute to a lack of hope for the future.

The next common factor determining the SRH was socioeconomic status. Our study revealed that there was a significant difference among different socioeconomic groups in terms of self-rated health as poor people reported a lower health status compared to better-off individuals. In comparison with people from lower economic background, being at the middle socioeconomic class or rich group reduces the chance of stating poor health by 0.52 and 0.40, respectively. Other studies have shown similar results and found that better economic position is associated with better SRH status (22–27). According to the study by Nejat in Tehran, the chance of stating bad SRH in people in the poorest quintile was 4.3 times more than the richest (28). The study done by Moor et al. on adolescence in European countries showed a strong relationship between socioeconomic indicators such as subjective social status and family influence (29). It should be noted that belonging to a worse socioeconomic condition means living with many limitations known as social determinants of health which can influence health negatively.

Like other studies, a negative association was found between suffering from underlying diseases and the status of SRH. In fact, it was the second factor with the highest effect on reporting a bad SRH. For example in an Iranian study, poorer SRH was significantly related to a higher prevalence of chronic illnesses (OR, 1.61), psychological health disorders (OR, 1.69), and dermatologic disorders (OR, 1.30) (10). Similarly, in the study conducted in southwest of Iran, the authors found that those who suffered the most from chronic or long-term illnesses, psychological health disorders, dermatologic or hearing disorders had higher chance of reporting poor SRH status (10). This association of reporting bad SRH and underlying and chronic diseases is much clearer as those with underlying diseases are suffering from physical health problems.

Considering the impact of education on SRH, our findings confirmed the findings of other studies which showed that individuals with higher education enjoy better status of self-rated health (20, 30). Similarly in the study of Nejat in Tehran the odds of assessing SRH as bad in people with no formal education was 10 times more than those with tertiary education (28). However, some of previous studies conducted among Indian and Chinese older population showed inverse association between education and SRH (23, 31). This can be due to the reason that old people suffer from physical and chronic diseases which can overshadow any kind of association between SRH and other demographic variables.

Although global evidence indicates that self-rated health (SRH) tends to decline with advancing age (32), this was not approved in this study. Although marital status was not included in this study, other similar studies from India and revealed that married persons had a lower chance of stating poor SRH in comparison with their unmarried counterparts (32, 33).

Like other studies in China (24) we found a significant relationship between unemployment and not having health insurance coverage with poor SRH in our study. Studies from USA, UK, and Brazil also proved a positive association between unemployment and self-rated poor health (34, 35). Unemployment can negatively affect the physical and mental dimensions of health which can be reflected as a bad SRH. Although, unemployment showed a significant association with poor SRH status, this variable as well as history of mental disorders, and loss of family members, did not have a significant effect in the multiple model.

What was interesting was the positive impact of health care utilization on SRH. People with recent utilization of health care over the last year had a higher chance of reporting better SRH. This may indicate that those with bad SRH are suffering from some medical needs which have not been met on time. So it can be inferred that access to health care services can help people to address their health problems. Regarding this fact, extending health insurance coverage and deepening the health benefit packages can increase financial access for the public to use health services when they need them. The same finding was also proven in the study conducted in Sanandaj, Iran (1).

### Study limitations and strengths

Due to the cross-sectional nature of the study, the observed associations cannot be considered causal. The current study was conducted in deprived regions, which limits its generalizability to other parts of the country. Also there are other variables that affect SRH among population but not investigated in this study. They include spiritual and religious backgrounds, participation in mediation, being migrant daily living activities (36). Another factor influencing SRH is living place (living in urban or rural areas) which was not included in this study (32). Despite this, the results of our study, for the first time provided a snapshot of how people in the west of Iran think of their SRH status and the main contributors. Enjoying a large sample size, a high rate of participation, and a population-based design are also other strengths of our study. The study also followed rigorous methodology and quality control measures to minimize errors during data collection and analysis.

## Conclusion

In summary, the present study presented evidence on SRH status and influencing factors in Ilam province, an isolated location in the country suffering from chronic unpleasant economic condition. According to the findings of our study, it is evident that feelings of hopelessness can significantly affect health, particularly among the poor. This implies that it can also affect other aspect of lives directly or indirectly. Therefore, further research is needed to find reasons behind hopelessness and recommend short and long term applicable solutions to bring hope back to society. Our results also revealed that individuals from underprivileged groups, those with low-education level, suffering from psychological health problems were more likely to assess their health as poor. These factors in turn are affected by many other causes, requiring further studies to identify the rooted causes and implementing multi-facet measures in various areas of the society. This requires devising corrective measures and effective public health policies to address causes and factors associated with poor SRH. Besides that, local health officials should allocate financial resources and other kinds of supportive initiatives to provide targeted support for those who are struggling with poverty and suffering chronic diseases.

## Data Availability

The raw data supporting the conclusions of this article will be made available by the authors, without undue reservation.
