# Supplementary material for: Self-rated health status and associated factors in Ilam, west of Iran: results of a population-based cross-sectional study
Source: Front Public Health. 2025 Jan 7;12:1435687. doi: 10.3389/fpubh.2024.1435687 (PMC11747038; doi:10.3389/fpubh.2024.1435687)
Supplement: Supplementary file 1 [file Table_1.DOCX]

**Supplement 1: used interview guide in present study**

The present questionnaire is about investigating the self-rated health in the general population of Ilam city, and the factors affecting this relationship. We request you to answer the questions with sincerity. It should be noted that all questionnaires are anonymous, and all the information in the questionnaire is considered confidential. The information will be analyzed in aggregate only. Voluntarily completing this questionnaire constitutes your consent to participate in this study.

**Demographic Characteristics**

1-race: Kurd Arab Lour Turk Persian other

2-Insurance: Yes No

3-Age: …………………. Yrs. old: 4-Weight: …………………. Kg 5-Height: …………………. cm

6-Gender: male female

7-Marital status: married single divorced widow

8-Education: Master's degree and above ☐ Bachelor's degree ☐ Associate Degree ☐ Diploma ☐ Under diploma

9-Employment status: student or student employee freelance retired housewife unemployed

10- Have you used any healthcare services in the past year, such as dentistry, surgery, or prescription medication?

Yes No

11- Have you seen a doctor in the last 6 months? Yes No

12-Family size: ……….... person

13-history of suffering from a specific disease -

Diabetes Blood pressure Asthma Cancer Cerebrovascular diseases mental disorder (depression, anxiety etc.) Cardiovascular diseases (anterior and cerebral) Kidney diseases (dialysis etc.) Other by mentioning the type of disease: .........

14- Economic characteristics: (Please select the checkbox next to each device that you have in your home)

Car Motorcycle Mobile phone Refrigerator Dishwasher Macrowave Laptop Vacuum cleaner Washing machine Internet access LCD TV

DVD player Going to a restaurant steam irons Home/house/flat

15-Have you ever smoked hookah for at least 6 weeks over a period of 6 months?

Yes No

16-Have you ever used alcohol at least once a month for a total of 6 months?

Yes No

17-Have you used illegal substances (opium and its derivatives, cannabis, marijuana, cigarettes, ecstasy, amphetamine, Ritalin) at least 6 months in total?

Yes No

18-During your lifetime, have you smoked at least one cigarette a day for at least 6 consecutive months or for a total of 180 cigarettes over any period of time?

Yes No

19-Has a member of your family ever died?

Yes No

20- Based on the overall situation in the country and society, do you have any hope for the future?

Very much Much Medium Little Very little

21-Have you ever lost your job?

Yes No

**Self-rated Health**

Dear participant, the following question is for self-evaluation of your health status. Please read the question below carefully and select one of the options.

In general, how would you rate your health at this moment?

Very good Good Average Bad Very bad
