# Supplementary material for: Self-rated health status and associated factors in Ilam, west of Iran: results of a population-based cross-sectional study
Source: Front Public Health. 2025 Jan 7;12:1435687. doi: 10.3389/fpubh.2024.1435687 (PMC11747038; doi:10.3389/fpubh.2024.1435687)
Supplement: Supplementary file 2 [file Table_2.DOCX]

Supplement table 1: description of study variables along with definitions and coding

| Variable | Definitions | Measuring | Coding |
| --- | --- | --- | --- |
| Age | Age of person according to self-reporting in years scale | Self-reporting | Continuous number |
| Gender | By phonotype and according to self-reporting | Self-reporting | Male=0; Female=1 |
| Race | Race of person according to self-reporting | Self-reporting | Other = 1; Kurd = 2 |
| Education level | Last educational degree according to self-reporting | Self-reporting | < Diploma =1; Diploma and Associate Degree = 2; ≥ Bachelor = 3 |
| Household size | number of members in family according to self-reporting | Self-reporting | Continuous number |
| Occupation | Jobs that person done for earing income or done as a regular action | Self-reporting | Student =1; Employed=2; Retired=3; Housekeeper or unemployed=4 |
| Health insurance | Any type of insurance that covers the whole or a part of the risk of a person incurring medical expenses | Self-reporting | Covered =1, Un-covered = 2 |
| Economic status | A wealth index that was constructed by asking about assets’ person | Self-reporting | Low =1; Middle=2; High=3 |
| Co-morbidities | Having at least on type of chronic disease including diabetes, hypertension, asthma, cancer, central nervous disorder, musculoskeletal disorder, cerebrovascular diseases and kidney diseases according to physician decision | Self-reporting | No=0; Yes=1 |
| Mental disorders | Having history of common mental disorders including depression, stress, anxiety, paranoid, schizophrenia and PTSD according to physician decision | Self-reporting | No=0; Yes=1 |
| Family member death history | Dying at least one member of family person including gather, mother, brother, sister, son and girl | Self-reporting | No=0; Yes=1 |
| Job loss history | Having history of job losing and being unemployment at least for 6 month | Self-reporting | No=0; Yes=1 |
| Hope for the future | How much you have hope to future according to current situation | Self-reporting | No; Little, Mild= 1; High, Very high= 2 |
| Use of health care service | Going to health care centers to getting health services at least one time during last year | Self-reporting | No=0; Yes=1 |
| Have a doctor visit | Going to hospitals or clinics to getting medical services at least one time during last year | Self-reporting | No=0; Yes=1 |
| Smoking | Have you ever smoked cigarettes in your lifetime? | Self-reporting | No=0; Yes=1 |
| Alcohol | Have you ever consumed alcohol in your lifetime? | Self-reporting | No=0; Yes=1 |
| Hookah | Have you ever smoked hookah in your lifetime? | Self-reporting | No=0; Yes=1 |
| Self-rated health | how would you rate your health? | Self-reporting | very poor, poor =1; fair =2; good, very good =3 |
